# Monitoring the Air Quality in an HVAC System via an Energy Harvesting Device

**DOI:** 10.3390/s23146381

**Published:** 2023-07-13

**Authors:** Corrado Boragno, Orazio Aiello, Daniele D. Caviglia

**Affiliations:** 1 Department of Physics (DIFI), University of Genova, 16146 Genova, Italy; 2 Department of Electrical, Electronics and Telecommunication Engineering and Naval Architecture (DITEN), University of Genova, 16145 Genova, Italy; orazio.aiello@unige.it (O.A.); daniele.caviglia@unige.it (D.D.C.)

**Keywords:** powering HVAC systems, battery-less air monitoring, air flux, wind energy harvesting, autonomous wireless sensor networks

## Abstract

The energy consumption of a heating, ventilation, and air conditioning (HVAC) system represents a large amount of the total for a commercial or civic building. In order to optimize the system performance and to increase the comfort of people living or working in a building, it is necessary to monitor the relevant parameters of the circulating air flux. To this end, an array of sensors (i.e., temperature, humidity, and CO_2_ percentage sensors) is usually deployed along the aeraulic ducts and/or in various rooms. Generally, these sensors are powered by wires or batteries, but both methods have some drawbacks. In this paper, a possible solution to these drawbacks is proposed. It presents a wireless sensor node powered by an Energy Harvesting (EH) device acted on by the air flux itself. The collected data are transmitted to a central unit via a LoRa radio channel. The EH device can be placed in air ducts or close to air outlets.

## 1. Introduction

The proliferation of energy consumption and CO2 emissions in the built environment has made energy efficiency and savings strategies a priority objective for energy policies in most countries. A clear example of this is the European Energy Performance of Buildings Directive (EPBD) [1]. Especially important has been the intensification of energy consumption in heating, ventilation, and air conditioning (HVAC) systems, which has now become almost essential in parallel to the spread of the demand for thermal comfort, considered a luxury not long ago. It is the largest energy end-use both in the residential and non-residential sectors: for dwellings, it represents about half of the energy consumption, more than doubling that for DHW (Domestic Hot Water) [2]. For non-domestic buildings, European government agencies estimate HVAC energy consumption to be around 48%, still lower than the 57% in the USA and similar to figures from other sources [3]. Regarding inside comfort, it is now clear that insufficient ventilation causes human-produced carbon dioxide to build up indoors, decreasing employee well-being and productivity substantially. With accurate CO_2_ measurements, both energy efficiency and employee well-being can be achieved simultaneously. The Federation of European Heating, Ventilation and Air Conditioning (REHVA) states that decreased ventilation lowers productivity, for example, typing speed, by 10%. The US Green Building Council commenced a meta-study in 2003 and concluded that the delivery of fresh air and reduced levels of pollutants improve productivity by 11%. Furthermore, the correct values of temperature, humidity, and CO_2_ percentage have been found to be crucial for the well-being of people working or living inside.

Air pollution is monitored by measuring the concentrations of different contaminants at a fixed site by employing precise but pricey equipment. Then, to monitor the number of pollutants and CO_2_ concentration, battery-operated or wired sensors are placed in different rooms or in strategic points of the HVAC plant (i.e., aeraulic ducts) that are difficult to access. Thus, the use of self-powered devices is the key element for effective and reliable pollution monitoring [4]. On this basis, a new trend has emerged in the use of readily available, portable sensors [5] that operate similarly to traditional battery-operated or wired sensors. In this framework, energy harvesters can provide the necessary power for these air quality monitoring devices, which must run continuously without human intervention [6,7,8,9,10,11,12,13,14,15,16,17]. This will meet a few goals of the United National 2030 *Agenda for Sustainable Development*, such as “Goals 7—Ensure access to affordable, reliable, sustainable and modern energy for all”; “Goals 11—Make cities and human settlements inclusive, safe, resilient and sustainable”; and “Goals 12—Ensure sustainable consumption and production patterns” [18].

The data provided by the sensors are then stored and transmitted to the cloud via in-place Wireless Sensor Networks (WSNs). These WSNs are generally more and more employed for the monitoring of the natural environment, transportation vehicles, industrial plants, and smart cities. Again, the feasibility of these WNS nodes is related to the possibility of enabling energy-autonomous systems. Even if the power demand from each node is low, usually in the range from µW to tens of mW, WSNs can contain a large number of nodes (e.g., hundreds) distributed over a wide area, making the node wiring complex, expensive, or completely impractical; moreover, disposable batteries are characterized by a high environmental impact and high maintenance costs.

In recent years, Energy Harvesting (EH) devices that are able to locally convert into electricity otherwise wasted forms of energy available in the surrounding environment have been widely investigated: sun, wind, vibrations, rainfalls, electromagnetic fields, and temperature gradients have been considered useful energy sources. EH devices can be applied in many contexts, like environmental monitoring [14,15,16,17], industrial plants [19], smart cities [20], and smart agriculture [21].

This paper focuses on a particular application of an EH device acted on by an air flux. In particular, the target is the monitoring of the air flux in an HVAC plant. The paper is organized as follows: In Section 2, the EH device is described. Then, in Section 3, the measurement results are reported. Finally, in Section 4, the conclusions are drawn.

## 2. Materials and Methods

### 2.1. The Energy Harvesting Device

The EH device introduced in this paper is a Fluttering Energy Harvester for Autonomous Powering built with Magnetic suspension (named FLEHAPMag hereafter). It is an updated version of the Fluttering Energy Harvester that restores force via elastomers or springs (with elastomeric suspension) presented in [22,23].

**Figure 1:**
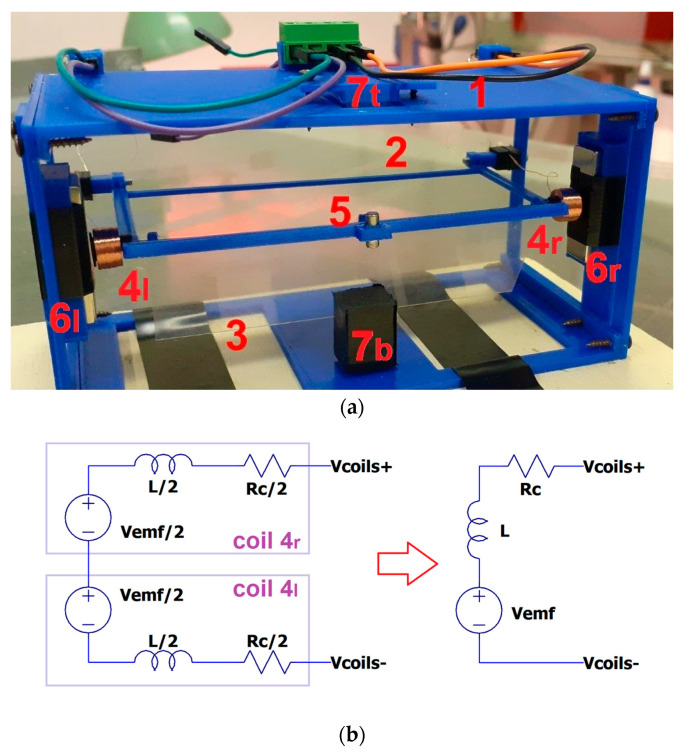
(**a**) A schematic view of the FLEHAPMag device: (1) main plastic structure; (2) oscillating plastic frame; (3) transparent foil wig; (4) coil; (5) moving magnet; (6) lateral magnets; (7) suspension magnets. (**b**) Equivalent model of its electric circuit: the three components of each coil are connected in series and add up as follows: $L=20\;\mathrm{mH},{\;R}_{C}=300\;\Omega$.

FLEHAPMag is schematically presented in Figure 1. It consists of a rigid rectangular U-shaped structure (2) hinged at one end to a frame (1). This structure features an axis around which a transparent foil wing (3) can freely rotate, a small permanent magnet (5), and two coils (on the left 4l and on the right 4r). The frame (1) houses two series of permanent magnets (on the left 6l and on the right 6r) facing the coils and two auxiliary magnets (on the top 7t and on the bottom 7b) with opposite polarization with respect to the small one (5). When wind (or, more generally, a gas in motion) impacts the device, part of the kinetic energy of the fluid is transferred to the wing, which exerts a fluid dynamic force on (2). The magnetic system (7t–5–7b) is adjusted to provide a restoring nonlinear force for the system, allowing the trigger of periodic oscillations of (2) to be activated: as a result, a current is induced in the coils moving in front of the permanent magnets (6l and 6r) due to Faraday’s effect.

The device is based on a well-known fluid dynamic effect named “flutter”, a dynamic instability of an elastic structure in a fluid flow, caused by positive feedback between the body’s deflection and the force exerted by the fluid flow. Structures exposed to aerodynamic forces—including wings and airfoils, but also chimneys and bridges—are designed carefully within known parameters to avoid flutter, but in the present case, it is exploited to harvest energy.

An equivalent model of the electrical part of the harvester is depicted in Figure 1b. $V_{\mathrm{emf}}$ is the electromotive force generated by the coils, L is the inductance of the two coils; Rc is their parasitic resistance, and $V_{\mathrm{coils}}={\;V}_{\mathrm{coils}+}$ − ${\;V}_{\mathrm{coils}-}$ is the differential output of the harvester (which in the case of no load, is equal to $V_{\mathrm{emf}}$).

A detailed analysis of the forces acting on the device is reported in [22]; only the main features of the problem are reported in the following:

- The resulting force is the sum of $F_{w}+F_{f}+F_{\mathrm{el}}+F_{\mathrm{EC}}$, where $F_{w}$ is the weight of the system, $F_{f}$ is the fluid force due to the air flux, $F_{\mathrm{el}}$ is the elastic restoring force, and $F_{\mathrm{EC}}$ is the force due to the electromagnetic coupling. These forces have a corresponding resulting momentum acting on the frame.
- $F_{\mathrm{EC}}$ represents the coupling between the mechanical and the electric parts. The current *I* in the coils obeys the following equation:
  $$L\dot{I}+\left(R_{C}+{\;R}_{L}\right)I=V_{\mathrm{emf}}$$
  where $R_{L}$ is the load resistance. Notice that the electromotive force $V_{\mathrm{emf}}$ can be written as $V_{\mathrm{emf}}=-{\Phi y}_{\mathrm{dot}}$, where Φ is the magnetic flux across the coils, and $y_{\mathrm{dot}}$ is the vertical velocity of the coils.
- The current I flowing in the coils induces a counteracting force on the frame $F_{\mathrm{EC}}=k\cdot I$, where k is a constant depending on the strength of the permanent magnets and on the dimensions of the coils.

**Figure 2:**
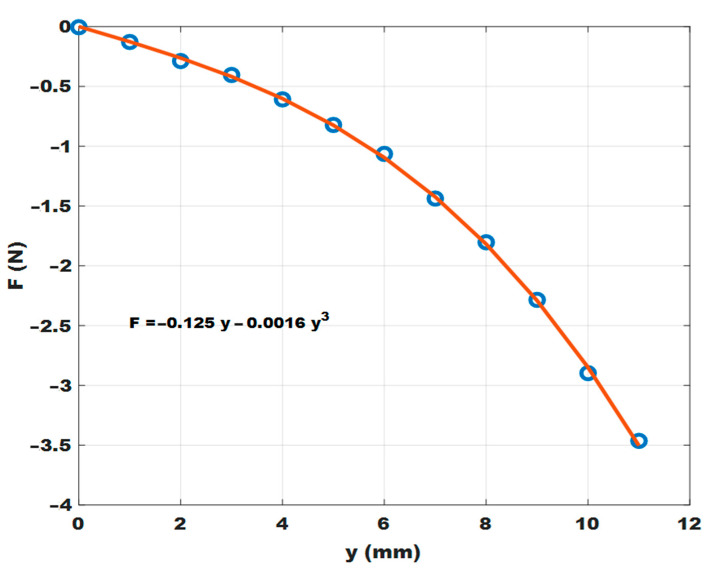
Simulation results for the pullback force as a function of the gap between the magnets 5 and 7t at each mm (blue circles) and best fitting curve value (red line).

The magnet system (7t–5–7b) provides a nonlinear pullback force, the intensity of which can be easily adjusted by varying the balance distance between the central magnet (5) and magnet pair (7t and 7b). In this way, it is possible to adjust the rate at which the system oscillations are triggered, adapting the device to different operating conditions. As an example, Figure 2 shows the result of a simulation obtained with Femm 4.2 software [24]. At y = 0 mm, the magnet (5 whose size length is 6 mm and outer diameter is 2 mm) is in equilibrium with respect to the two magnets (7b,t of size 5 × 5 × 5 mm^3^ each) spaced 12 mm apart. These values are quite similar to those of the actual arrangement. As an example, considering the distance between the central magnet (5) and the top magnet (7t) as the latter decreases, the pullback force increases following a cubic law, as shown in Figure 2.

The fluttering effect is characterized by having a trigger fluid velocity, which depends on the elasto-mechanical characteristics of the system. In the present configuration, the system starts to oscillate when the flux velocity is around 2.7 m/s and stops at 6 m/s. With respect to the system reported in [22], the current configuration is more robust and versatile, reducing the probability of failure and therefore increasing the lifetime of the device.

The supporting structures (1 and 2 in Figure 1) were made using a 3D printer (Sharebot 43), typically in PLA or ABS. The overall dimensions of the device are around 10 cm × 10 cm × 12 cm. The wing is a polyethylene foil with a chord of 30 mm and a span length of 95 mm (thickness 0.1 mm). The magnets were made in neodymium (NdFeB) with a disc or block shape [25]. Each coil has an external diameter of 9 mm, internal diameter of 5 mm, and height of 5 mm.

**Figure 3:**
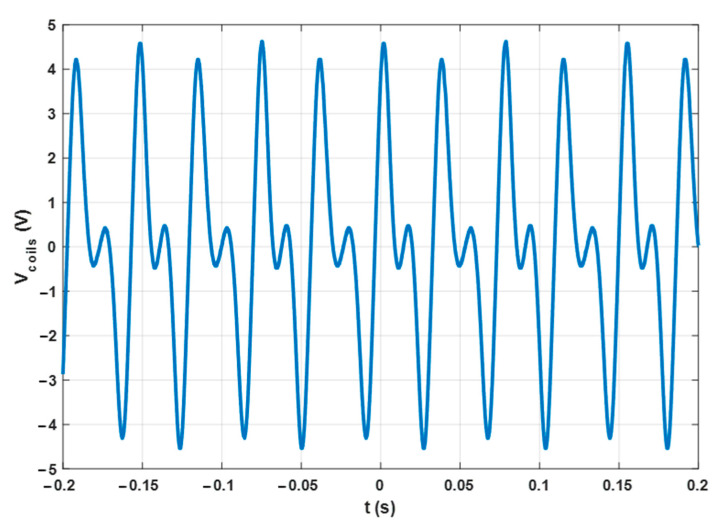
Measured differential output of the harvester $V_{coils}=V_{coils+}$ − $\;V_{coils-}$ (as defined in Figure 1b) versus time in open circuit condition. Air speed 3 m/s.

When the system is activated with no load (open circuit), the voltage across the coils appears as depicted in Figure 3. The oscillation typically features a frequency of around 13 Hz and a peak-to-peak value of around 9 V with an air speed of 3 m/s. These values depend not only on the airspeed but also on the mechanical properties of the device, like the mass distribution, wing area, and magnetic spring strength. Therefore, it is easy to tune the electrical output to meet the specific conditions of the ambiance where the device is deployed. In fact, the airspeed in an HVAC plant can be different in different parts of the system. The amount of power that can be extracted in the practical case is in the range of a few tens of milliwatts, more than enough to provide sufficient energy for the Wireless Sensor Node, as is demonstrated below.

In the scientific literature, many devices based on the interaction between the air flux inside the ducts of an HVAC plant and an aeroelastic structure are described [26,27,28], mainly based on a piezoelectric cantilever. The device explored in this paper has comparable performance in terms of power output, especially at low flow rates, and the advantage of little disturbance of the airflow, allowing several devices to be used in series.

### 2.2. Architecture of the Sensor Node

When designing the architecture of the sensor node, one important point to observe is that, when the EH is connected to a load, an electromagnetic brake effect occurs due to the currents flowing in the coils [29,30]. Consequently, the maximum power attainable depends on the combination of three aspects: the wind speed, the electrical source impedance, and the load current. Consequently, proper MPPT techniques have to be adopted for this purpose. However, one has to carefully design the node architecture to limit as much as possible the power consumption for both acquiring and transmitting sensory data.

The first point concerns the choice of radio protocol to adopt: it is worth noting that ours is simply an implementation example, and certainly, other solutions can be considered [31]. In particular, the main competitors seem to be LoRa [32] and Mioty [33], while the LoraWAN network was discarded because its handshaking protocol between the node and the gateway needs several seconds to be completed, resulting in excessive energy requirements [34]. As a result, the LoRa technology has been chosen., also because of its ability to operate in difficult scenarios, such as long-distance or out-of-line-of-sight links, with little energy requirements [32]. Notice that also Mioty represents a possible alternative and can be considered for future implementation.

The conditioning of the signal produced by the EH device (FLEHAPMag) is entrusted to an integrated specialized energy management IC (E-peas AEM30940 [35]), which uses an MPPT algorithm to charge a 15 mF capacitor used as an energy reservoir.

The fact of using a capacitor makes it possible not to use rechargeable batteries and consequently lowers both the construction and the management costs of the system. The charge stored in the capacitor feeds a Texas Instruments TPS63900 high-efficiency synchronous buck-boost converter [36], which provides the power supply to an Adafruit Feather M0 RFM96 LoRa Radio (433 MHz) [37]. The latter is equipped with an ATSAMD21G18 ARM Cortex M0 MCU [38] and an SX1276 LoRa transceiver [39]. The Arduino IDE [40] was used to develop the firmware. The operational management of the overall system relies on a Texas Instruments TPL5110 Nano-Power System Timer [41]. It is enabled by the AEM30940 when the voltage on Cstorage exceeds 3.8 V. In this condition, the timer enables the activation of the TPS63900 converter and the Feather module at one-minute intervals, and this is more than sufficient for HVAC control application.

The MCU then runs a short program that reads the data from the temperature and humidity sensors, prepares the radio packet, activates the transceiver for the RF transmission, and finally resets the timer. When the “done” signal is sent by the MCU to the timer, the latter de-activates the DC/DC converter, so the Feather module is turned off, and the power consumption is reduced to be lower than 500 nA, due to the timer and the resistive divider used for detecting the voltage across the Cstorage.

**Figure 4:**
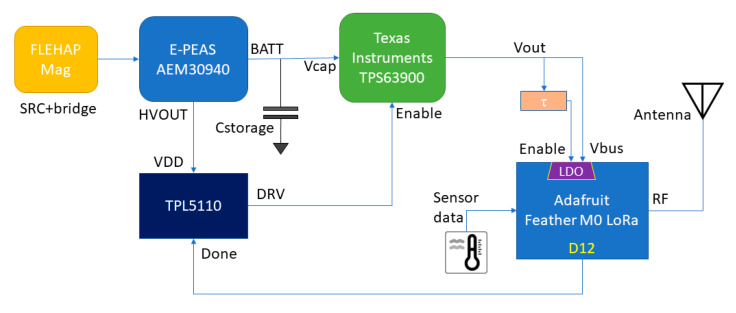
Block diagram of the sensor node architecture.

In Figure 4, a simplified block diagram of the overall circuit is reported where some synchronization signals are highlighted. The base station was implemented with a companion Adafruit Feather M0 LoRa board connected to a PC.

### 2.3. Implementation Insights

The specific goal of this work is to match the FLEHAPMag capabilities in terms of electric energy production in the specific application case, with the energy budget needs of the electronics for a reliable level of operation.

**Figure 5:**
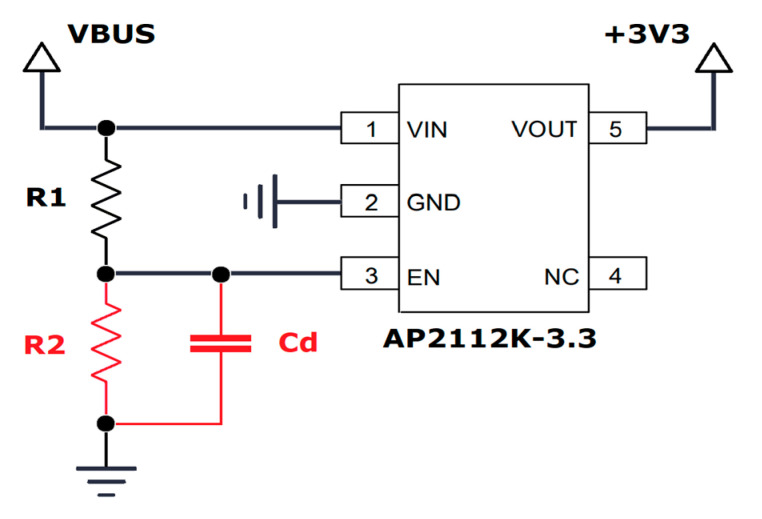
Schematics of the power regulation circuit of the Adafruit Feather board, arranged to introduce the τ delay. The original components are drawn in black, while the ones added to delay the power-up are depicted in red (R1 = R2 = 100 kΩ, Cd = 47 nF). The signals VBUS and +3V3 are named here after the original schematics provided by Adafruit [37], and they correspond to Vout and Vcc, respectively, in Figure 6.

**Figure 6:**
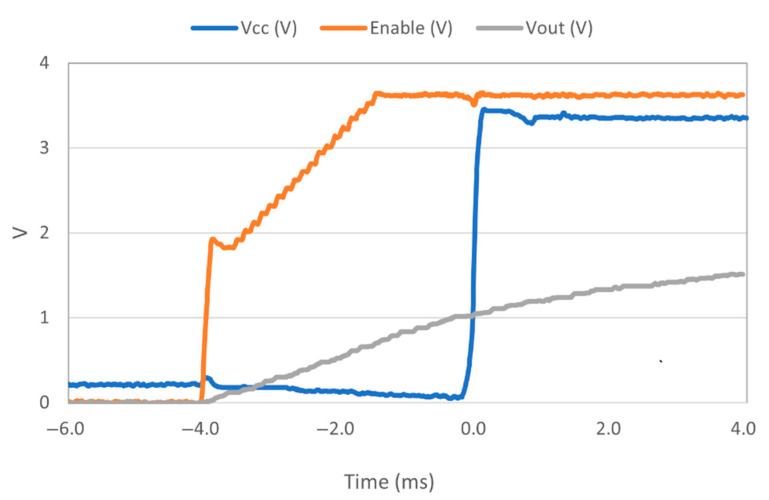
Measured power-up sequence: the green line is the output voltage (Vout) of the TPS63900 converter, which starts its rising progression once activated by the TPL5110 timer; the orange line is the delayed Enable input of the AP2112K-3.3 LDO; and the blue line is the V_CC_ of the board, powering both the MCU and the LoRa transceiver module.

The first important aspect concerns the behavior of the various circuits when switched on. In particular, within the ATSAMD21G18 MCU, the integrated Power-on Reset (POR) circuitry monitoring the analog supply voltage requires a minimum rise rate [38]. Even if its value is not specified on the datasheet, the rise time of the TPS63900 converter does not guarantee a proper power-up sequence [36]. To deal with this issue, the Enable input of the AP2112K-3.3 LDO [42] (which is used on the Adafruit Feather board to regulate the 3.3 V power line) has been exploited. By default, this input is tightened to the external voltage input of the board using a 100 kΩ resistor. An additional external resistor of the same value connected to the ground, with a 47 nF capacitor in parallel, introduces a delay of an approximate duration of 4 ms between the external supply and the activation of the LDO (as depicted in Figure 5). It is represented as the “τ” block in Figure 4. The final behavior that proved effective is illustrated in Figure 6. A short note should also be made regarding the V_CAP_ monitoring: a voltage divider with a total resistance of 12.2 MΩ was used to allow this operation with negligible consumption. 

The most important point to consider is limiting of the power consumption as much as possible and, consequently, the energy requirements, specifically as a function of the transmission parameters. To this end, it is advisable to shorten the time interval in which the Adafruit board remains on as much as possible. In this respect, the first important point is to limit the board startup time once it is powered up. This can be achieved by operating with the internal clock, instead of using the external (and more precise) 32.768 kHz crystal clock: in fact, the higher Q of the latter involves a much longer startup time. In the Arduino environment, this can be accomplished by adding the “DCRYSTALLESS” flag to the compiler command line [43]. In addition, referring to the application scenario detailed in the following section, some remarks have been drawn. In fact, considering the LoRa physical layer [32], two main parameters affect the energy needs: the Spreading Factor (SF) ranging from 7 to 12 and the output RF power, which, in principle, can span from +5 dBm to +23 dBm with the adopted transceiver [39]. Anyhow, it should be limited to a maximum EIRP (Effective Isotropic Radiated Power) of +10 dBm due to the regional limitations recommended for Europe [44]. A maximum duty cycle recommendation of 10% is also given [44]. Notice that in the explored case, the payload length is 6 bytes, carrying two 12-bit numbers, coded as ASCII characters.

Based on the characteristics of the building in which the experimental validation was conducted, the frugal combination of SF = 7 and Pout = 5 dBm has been set as the initial condition. The further parameters Bandwidth and Coding Rate were fixed to 125 kHz and 4/8 for all presented measurement validations. The capacitor was charged to a sufficiently high voltage level, and the wind was stopped when needed, leaving the electronics working only thanks to the energy stored in the capacitor. 

**Figure 7:**
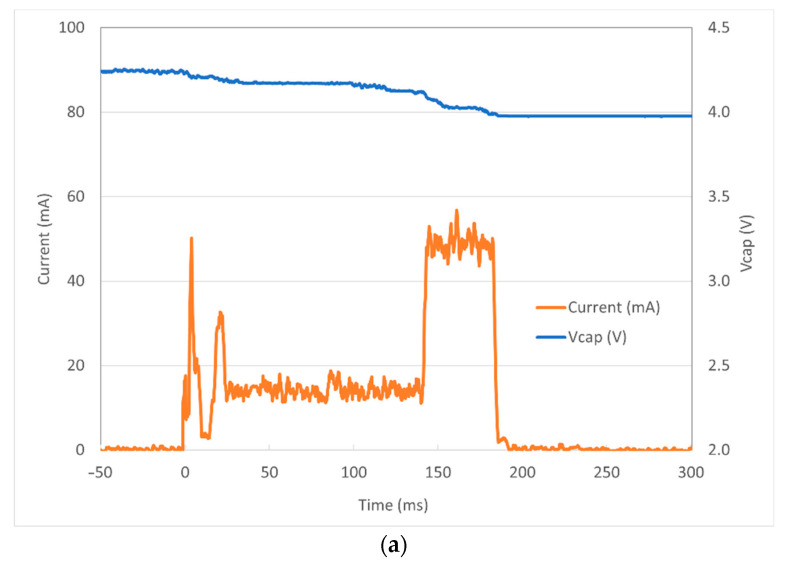

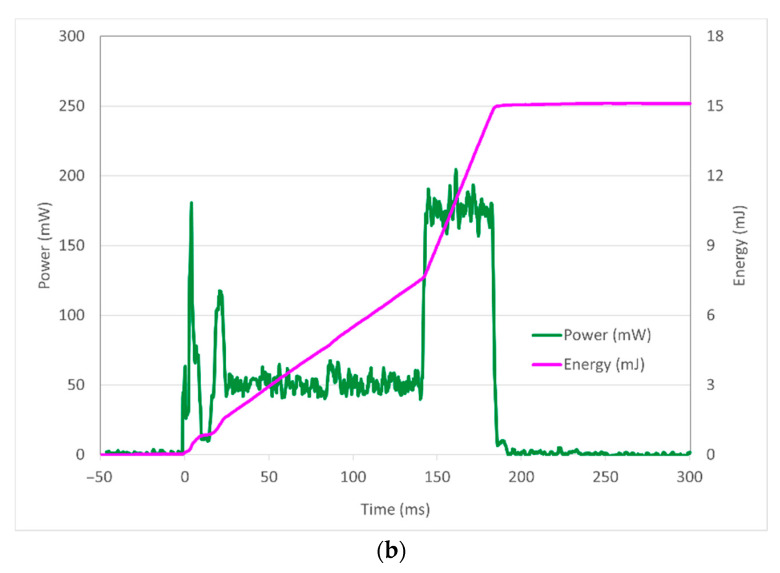
(**a**) Current delivered to the TPS63900 unit and capacitor voltage V_CAP_; (**b**) corresponding power and energy supplied to the Adafruit board.

The energy consumption signals corresponding to a single-packet transmission are shown in Figure 7. The initial voltage on the capacitor is V_CAP_(0) = 4.24 V, which corresponds to a stored energy of 134.9 mJ. At the end of the transmission, V_CAP_(0.18) = 3.98 V, which corresponds to a stored energy of 118.6 mJ. The energy supplied by the capacitor is consequently 16.4 mJ. This needs to be compared with the energy delivered to the Adafruit board, depicted in Figure 7b. In this figure, the power is obtained by multiplying the current in Figure 7a by the operating voltage of 3.6 V generated by the TPS63900E board, and the energy is determined by integrating the power over time. The interval ranging from 0 to about 142 ms corresponds to the sequence of tasks operated by the board, namely, the setup, transceiver configuration, sensor reading, and packet preparation. The energy needed for such operations sums up to about 7.6 mJ. The interval from 142 to 180 ms corresponds to the RF transmission. The current increases up to over 50 mA, the power exceeds 180 mW, and the energy consumption approximates 7.5 mJ, leading to a total of about 15.1 mJ. Considering the 16.4 mJ supplied by the capacitor, the derived efficiency of the power supply based on the TPS63900E chip reaches about 92%, which can be considered very satisfying and corresponds to the specification on its datasheet [36]. 

**Figure 8:**
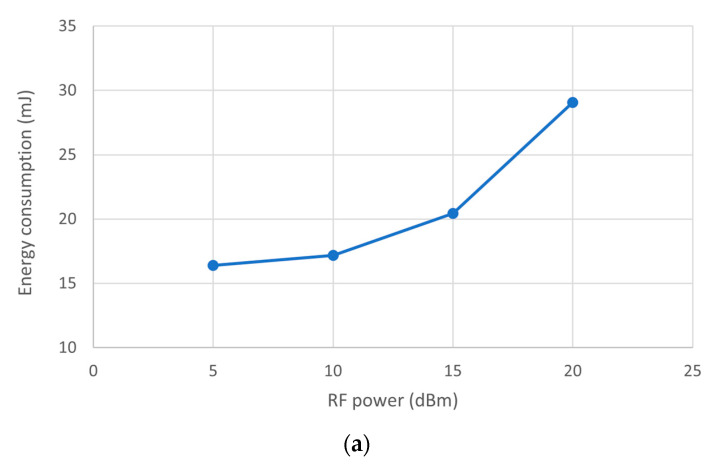

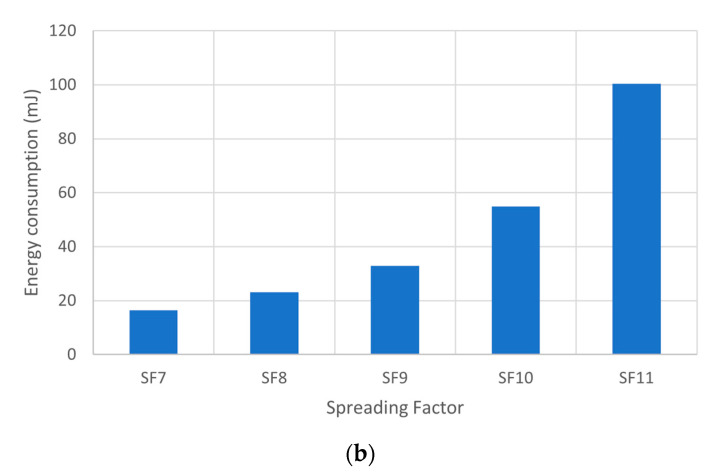
Energy consumption needed to transmit a packet (**a**) versus RF output power setting with Spreading Factor SF = 7, Coding Rate CR = 4/8, and bandwidth BW = 125 kHz and (**b**) versus the Spreading Factor settings for Pout = 5 dBm, CR = 4/8, and BW = 125 kHz.

It is worth considering the possible extension to different application scenarios, such as in wider buildings and different propagation conditions, which may require more robust RF communication configurations. An extensive study of all the possible configurations does not fall within the scope of this article: only a few examples and some considerations and suggestions have been presented. First, let us consider the possibility of changing the transmitting power. By repeating the same experiment in Figure 7 with different values, the curve represented in Figure 8a has been obtained. The second aspect to highlight is the dependence of the energy requirement on the Spreading Factor: Figure 8b reports the results of some experiments; it is worth noting that the case SF = 12 is not reported because there is not enough energy available to transmit even a single packet. It is worth considering the possible extension to different application scenarios, such as in wider buildings and different propagation conditions, which may require more robust RF communication configurations.

An extensive study of all the possible configurations does not fall within the scope of this article: only a few examples and some considerations and suggestions have been presented. First, let us consider the possibility of changing the transmitting power. By repeating the same experiment in Figure 7 with different values, the curve represented in Figure 8a has been obtained. The second aspect to highlight is the dependence of the energy requirement on the Spreading Factor: Figure 8b reports the results of some experiments; it is worth noting that the case SF = 12 is not reported because there is not enough energy available to transmit even a single packet.

To define how much energy is effectively available in the capacitor the minimum operating voltage of the timer and the buck/boost devices (that is 1.8 V) should be considered. This level should not be reached during normal operation; otherwise, the power supply system will fail. Consequently, considering a maximum voltage reachable at the capacitor of V_CAP_ = 5 V (which corresponds to 187.5 mJ in the current implementation) and the energy stored at 1.8 V of 24.3 mJ, the maximum energy budget available is 163 mJ. For example, the ability to transmit about ten packets on a single charge, which can be useful for the warning of any malfunction of the fan assembly has been set. The actual number of packets that can be transmitted with a single charge in the absence of airflow varies with the parameter configuration: for each use case, a tradeoff should be found among the storage capacitance, the payload length, the interval between transmissions, the LoRa parameter configuration, and the number of packets to be delivered with a single charge without wind.

## 3. Measurement Results

To demonstrate the soundness of the approach, a system with off-the-shelf components has been built. In particular, the 2AAAEM30940C0015 Evaluation Board from e-Peas and the TPS63900EVM Evaluation Module from Texas Instruments has been used.

**Figure 9:**
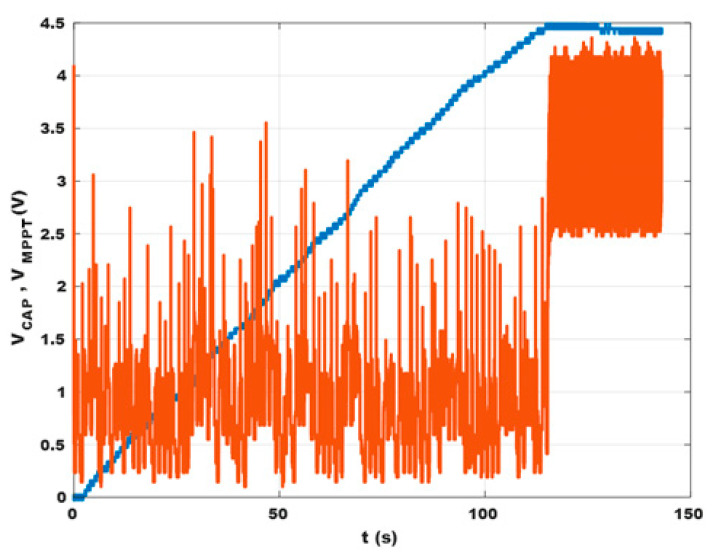
Voltage on the storage capacitor V_CAP_ (blue) and output voltage of the MPTT circuit V_MPPT_ (orange) versus time.

**Figure 10:**
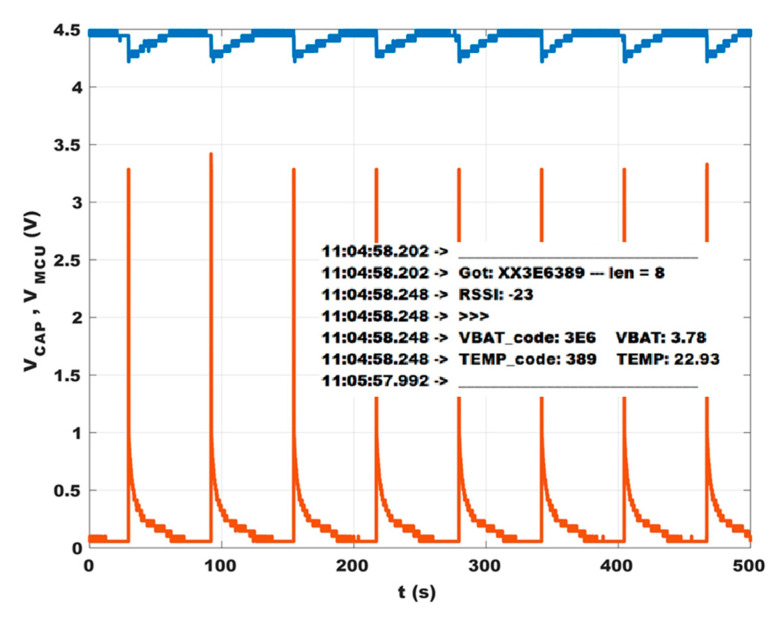
Voltage on the storage capacitor V_CAP_ (in blue) and the input voltage on the MCU V_MCU_ (in orange). In the inset, a test message is shown, as it appears at the base station: it includes a rough message containing the node identifier (“XX”), the voltages of the storage capacitor (named here VBAT), the temperature reading in degree Celsius, the RSSI detected by the receiver, and the time stamp added by the PC.

In order to have a point of reference to characterize the behavior of the system, the EH device with its signal conditioning circuit (without any load) has been firstly inserted in an open-end wind tunnel. The airspeed was set to 3 m/s, a typical value for an HVAC plant. The voltage on the storage capacitor V_CAP_ and the output of the MPTT circuit V_MPPT_ as a function of time have been measured (Figure 9). V_CAP_ increased roughly linearly, reaching 3.8 V after 90 s and the maximum value of 4.5 V after 115 s. Since C = 15 mF, the average power harvested was 1.1 mW. The MPPT algorithm stops when V_CAP_ = 4.5 V. In the same conditions, it is possible to seek the optimal resistive load as described in [45]: with the reported setup, it was found to be R_LOAD_ = 1 kΩ. The corresponding maximum power yielded by the EH was 1.4 mW. So, the average efficiency shown by the e-Peas IC until reaching the maximum output voltage was around 80%. By connecting the DC/DC converter and the LoRa card to the capacitor, data collection and transmission can begin: with each data reading and transmission, part of the energy stored in the capacitor is used, as shown in Figure 10. In this example, for demonstration purposes, a low-cost temperature sensor (model MCP9700A [46]) was used. The MCU reads the sensor output voltage, and V_CAP_ codes both as a three-digit HEX number, composes the final payload by adding a 2-byte node identifier, represents the three fields via ASCII characters, and transmits the data every 60 s. Each reading/transmission featured an energy cost of around 15 mJ, setting the radio transmitter power at 5 dBm. This power was verified to be sufficient to cover a range of about 40 m inside a building, with walls, doors, etc.

After a transmission, V_CAP_ returned to its maximum value approximately in 25 s, thus enabling a shorter transmission rate or possibly a higher power consumption, e.g., increasing radio power or using more sensors. 

**Figure 11:**
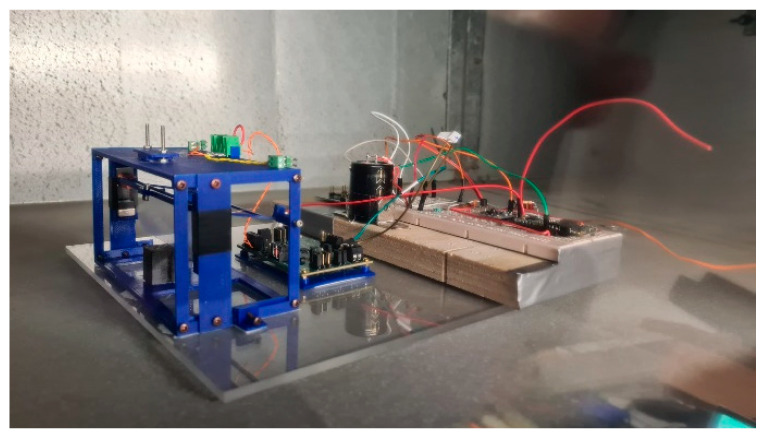
The EH device with electronics in an HVAC plant.

After the characterization of the wind tunnel, the system has been placed in an operating HVAC plant serving a gym. The device was placed in the exhaust part of the aeraulic ducts (Figure 11). The airspeed fluctuated around 2.7 m/s, and it was characterized by large turbulence, as expected.

**Figure 12:**
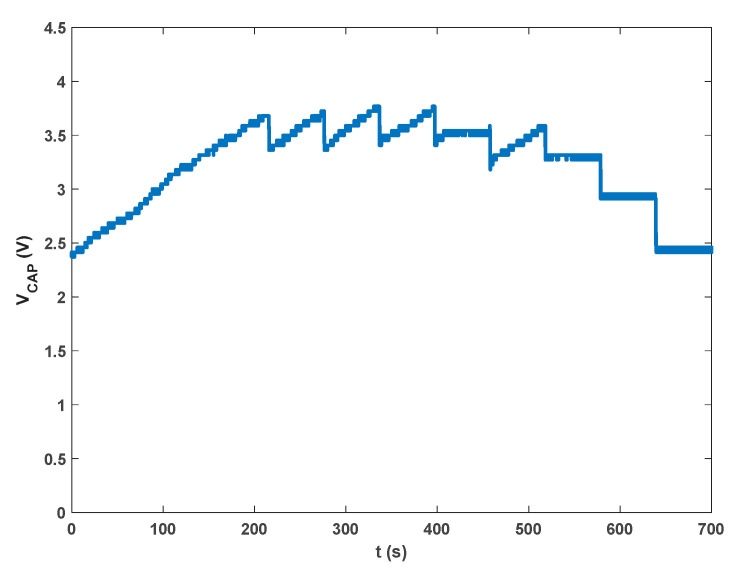
Voltage on the storage capacitor V_CAP_ versus time recorded in the operating HVAC plant.

The EH device worked well in real conditions, as shown in Figure 12. It is shown that the system started from an initial condition of V_CAP_ = 2.4 V, which is not sufficient to activate the operation. As V_CAP_ reached 3.8 V, it began to work. After this point, the V_CAP_ time behavior is similar to that reported in Figure 9. The main effect of the turbulence and of the fluctuating airspeed is a less regular oscillation of the EH device, causing a longer time to charge the capacitor from zero and a longer recovery time after data transmission. After 400 s, the air flux was stopped, but the energy in the capacitor was sufficient to allow two more transmissions before the system entered the quiescent state.

## 4. Discussion and Conclusions

The FLEHAPMag device acted on by air flux can be conveniently used to monitor the air parameter in an HVAC plant has been demonstrated. Due to its versatility, the EH device can be placed in many different sectors of an HVAC plant (i.e., positioned on the output of a fan coil). Since the device is not based on rotating blades, as usual in large wind turbines, the air flux is poorly disturbed at the exit; thus, it is possible to place two or more devices in close contact to obtain more power if needed. The time needed to completely charge the super-capacitor is around 115 s (with an air speed of 3 m/s), and each transmission of the sensor data (temperature) requires only 5% of the accumulated charge. In a real environment, this rate is probably not necessary: in a more realistic situation, a data refreshing rate close to 300 s could be obtained under the same conditions with a capacitor of the order of 40 mF. With these numbers, the energy available would be around 400 mJ for a cycle, and then more energetically demanding sensors could be powered using a single FLEHAPMag device. Finally, the turbulence inside a real aeraulic duct slightly deteriorates the performance of the device. However, this is not a critical issue: a different cage hosting the device and lowering the turbulence can be easily built (i.e., it can be inserted at the front side an array of cylindrical tubes to regularize the air flux).

Future work will be devoted to integrating the electronics into a smaller volume, adding more sensors (e.g., for CO_2_ concentration), and possibly further improving the efficiency of the system.

## Data Availability

Not applicable.

## References

[1] European Commission—Energy Energy Performance of Buildings Standards. https://energy.ec.europa.eu/.

[2] U.S. Department of Energy (2020). Residential Energy Consumption Survey (RECS). https://www.eia.gov/consumption/residential/data/2020/.

[3] U.S. Department of Energy (2018). Commercial Buildings Energy Consumption Survey (CBECS). https://www.eia.gov/consumption/commercial/data/2018.

[4] Cetinkaya O., Zaghari B., Bulot F.M.J., Damaj W., Jubb S.A., Stein S., Weddell A.S., Mayfiel M., Beeby S. (2021). Distributed Sensing with Low-Cost Mobile Sensors Toward a Sustainable IoT. IEEE Internet Things Mag..

[5] Bulot F.M.J., Russell H.S., Rezaei M., Johnson M.S., Ossont S.J.J., Morris A.K.R., Basford P.J., Easton N.H.C., Foster G.L., Loxham M. (2020). Laboratory comparison of low-cost particulate matter sensors to measure transient events of pollution. Sensors.

[6] Touati F., Legena C., Galli A., Crescini D., Crescini P., Mnaouer A.B. (2014). Renewable energy-harvested sensor systems for air quality monitoring. Proceedings of the 2014 26th International Conference on Microelectronics (ICM).

[7] Grossi M. (2021). Energy Harvesting Strategies for Wireless Sensor Networks and Mobile Devices: A Review. Electronics.

[8] Panayanthatta N., Clementi G., Ouhabaz M., Costanza M., Margueron S., Bartasyte A., Basrour S., Bano E., Montes L., Dehollain C. (2021). A Self-Powered and Battery-Free Vibrational Energy to Time Converter for Wireless Vibration Monitoring. Sensors.

[9] Mishu M.K., Rokonuzzaman M., Pasupuleti J., Shakeri M., Rahman K.S., Binzaid S., Tiong S.K., Amin N. (2021). An Adaptive TE-PV Hybrid Energy Harvesting System for Self-Powered IoT Sensor Applications. Sensors.

[10] Zhang Z., Wang H., Yang C., Sun H., Yuan Y. (2023). Vibration Energy Harvester Based on Bilateral Periodic One-Dimensional Acoustic Black Hole. Appl. Sci..

[11] Li W., Leng B., Hu S., Cheng X. (2023). Improving the Output Efficiency of Triboelectric Nanogenerator by a Power Regulation Circuit. Sensors.

[12] Wei Y., Duan J., Jing H., Yang H., Deng H., Song C., Wang J., Qu Z., Zhang B. (2022). Scalable, Dual-Band Metasurface Array for Electromagnetic Energy Harvesting and Wireless Power Transfer. Micromachines.

[13] Carter J., Rahmani A., Dibaj M., Akrami M. (2023). Rainwater Energy Harvesting Using Micro-Turbines in Downpipes. Energies.

[14] La Rosa R., Dehollain C., Livreri P. (2020). Advanced Monitoring Systems Based on Battery-Less Asset Tracking Modules Energized through RF Wireless Power Transfer. Sensors.

[15] La Rosa R., Dehollain C., Burg A., Costanza M., Livreri P. (2021). An Energy-Autonomous Wireless Sensor with Simultaneous Energy Harvesting and Ambient Light Sensing. IEEE Sens. J..

[16] Pop-Vadean A., Pop P.P., Latinovic T., Barz C., Lung C. (2017). Harvesting energy and sustainable power source, replace batteries for powering WSN and devices on the IoT. Proceedings of the IOP Conference Series: Materials Science and Engineering.

[17] Jiang D., Lian M., Xu M., Sun Q., Xu B.B., Thabet H.K., El-Bahy S.M., Ibrahim M.M., Huang M., Guo Z. (2023). Advances in triboelectric nanogenerator technology—Applications in self-powered sensors, Internet of things, biomedicine, and blue energy. Adv. Compos. Hybrid Mater..

[18] UN The 17 Goals. Sustainable Development Goals. Retrieved 10 August 2022. https://sdgs.un.org/goals.

[19] Hosseinkhani A., Younesian D., Eghbali P., Moayedizadeh A., Fassih A. (2021). Sound and vibration energy harvesting for railway applications: A review on linear and nonlinear techniques. Energy Rep..

[20] Liu L., Guo X., Lee C. (2021). Promoting smart cities into the 5G era with multi-field Internet of Things (IoT) applications powered with advanced mechanical energy harvesters. Nanoenergy.

[21] Xia L., Ma S., Tao P., Pei W., Liu Y., Tao L., Wu Y. (2023). A Wind-Solar Hybrid Energy Harvesting Approach Based on Wind-Induced Vibration Structure Applied in Smart Agriculture. Micromachines.

[22] Boccalero G., Olivieri S., Mazzino A., Boragno C. (2017). Power harvesting by electromagnetic coupling from wind-induced limit cycle oscillations. Smart Mater. Struct..

[23] Olivieri S., Boccalero G., Mazzino A., Boragno C. (2017). Fluttering conditions of an energy harvester for autonomous powering. Renew. Energy.

[24] Meeker D. Finite Element Method Magnetics. www.femm.info.

[25] www.supermagnete.it.

[26] Petrini F., Gkoumas K. (2018). Piezoelectric energy harvesting from vortex shedding and galloping induced vibrations inside HVAC ducts. Energy Build..

[27] Wang J., Tang L., Zhao L., Zhang Z. (2019). Efficiency investigation on energy harvesting from airflows in HVAC system based on galloping of isosceles triangle sectioned bluff bodies. Energy.

[28] Han N., Zhao D., Schluter J.U., Goh E.S., Zhao H., Jin X. (2016). Performance evaluation of 3D printed miniature electromagnetic energy harvesters driven by air flow. Appl. Energy.

[29] Haidar M., Chible H., Boragno C., Caviglia D.D. (2021). A Low Power AC/DC Interface for Wind-Powered Sensor Nodes. Energies.

[30] Boccalero G., Boragno C., Caviglia D.D., Morasso R. (2016). FLEHAP: A Wind Powered Supply for Autonomous Sensor Nodes. J. Sens. Actuator Netw..

[31] Orfanos V.A., Kaminaris S.D., Papageorgas P., Piromalis D., Kandris D. (2023). A Comprehensive Review of IoT Networking Technologies for Smart Home Automation Applications. J. Sens. Actuator Netw..

[32] Semtech What Is LoRa?. https://www.semtech.com/lora/what-is-lora.

[33] MIOTY Alliance e.V Mioty Technology. https://mioty-alliance.com/miotytechnology/.

[34] LoRa Alliance What Is LoRaWAN® Specification. https://lora-alliance.org/about-lorawan/.

[35] AEM30940-RF-Vibration Energy Harvesting Datasheet, E-Peas Semiconductor. https://e-peas.com/wp-content/uploads/2022/09/e-peas-AEM30940-datasheet-RF-Vibration-energy-harvesting-09-22.pdf.

[36] Texas Instruments TPS63900 1.8-V to 5.5-V, 75-nA IQ Buck-Boost Converter with Input Current Limit and DVS. https://www.ti.com/product/TPS63900?qgpn=tps63900.

[37] Adafruit Adafruit Feather M0 RFM96 LoRa Radio—433MHz—RadioFruit. https://www.adafruit.com/product/3179.

[38] Microchip SAM D21/DA1 Family Datasheet. https://ww1.microchip.com/downloads/en/DeviceDoc/SAM_D21_DA1_Family_DataSheet_DS40001882F.pdf.

[39] Semtech SX1276/77/78/79—137 MHz to 1020 MHz Low Power Long Range Transceiver Datasheet. https://semtech.my.salesforce.com/sfc/p/#E0000000JelG/a/2R0000001Rbr/6EfVZUorrpoKFfvaF_Fkpgp5kzjiNyiAbqcpqh9qSjE.

[40] Overview of the Arduino IDE 1. https://docs.arduino.cc/software/ide-v1/tutorials/Environment.

[41] Texas Instruments TPL5110 Nano-Powered System Timer with MOS Driver and Manual MOSFET Power ON. https://www.ti.com/product/TPL5110.

[42] Diodes AP2112 600 mA CMOS Ldo Regulator with Enable Datasheet. https://www.diodes.com/assets/Datasheets/AP2112.pdf.

[43] Arduino Forum Arduino Zero without an External 32 khz Crystal. https://forum.arduino.cc/t/arduino-zero-without-an-external-32khz-crystal/625614/2,.

[44] CEPT/ERC Recommendation 70-03, 11 February 2022. https://docdb.cept.org/download/3700.

[45] Boccalero G., Boragno C., Morasso R., Caviglia D.D. A Sensor Node Driven by Air Flow. Proceedings of the 2017 New Generation of CAS (NGCAS).

[46] Microchip MCP9700—Low-Power Linear Active Thermistor IC. https://www.microchip.com/en-us/product/MCP9700.

